# Interpretable Small-Sample Hyperspectral Phenotyping of Wheat Protein Fractions via Order-Optimized Derivative Preprocessing and TabPFN

**DOI:** 10.3390/foods15193507

**Published:** 2026-10-01

**Authors:** Zihao Li, Zhaoyang Chen, Yuxing Wu, Meng Fang, Bolin Duan, Weilong Qin, Binhui Liu, Bo Li, Wenying Zhang

**Affiliations:** 1 Institute of Dryland Farming, Hebei Academy of Agriculture and Forestry Sciences, Hengshui 053000, China; 2 College of Plant Protection, Hebei Agricultural University, Baoding 071001, China; 3 Institute of Plant Protection, Hebei Academy of Agriculture and Forestry Sciences, Baoding 071001, China

**Keywords:** whole-wheat flour, Osborne protein fractions, hyperspectral imaging, derivative-order optimization, TabPFN, explainable machine learning

## Abstract

Rapid phenotyping of water- and salt-soluble wheat protein fractions can support grain quality evaluation and breeding. Whole-wheat flour from water-nitrogen and water-fertilizer trials was used to predict measured albumin and globulin contents and their derived sum, termed the metabolic-protein sum (albumin + globulin). After systematically comparing various preprocessing, wavelength selection, and regression approaches, a framework combining order-optimized derivative preprocessing (ODP), a hybrid feature-selection approach based on minimum redundancy maximum relevance (MRMR) and recursive feature elimination (RFE) and a tabular prior-data fitted network (TabPFN) regressor provided consistently strong test-set performance. The optimal derivative orders were 1.0 for albumin and the metabolic-protein sum and 0.8 for globulin. The ODP-MRMR-RFE-TabPFN framework retained 147, 46, and 70 wavelengths for albumin, globulin, and the metabolic-protein sum, yielding test set coefficient of determination (R^2^) values of 0.847, 0.693, and 0.850; root mean square error (RMSE) values of 3.073, 0.951, and 3.368 mg g^−1^; and residual prediction deviation (RPD) values of 2.554, 1.804, and 2.586, respectively. Leave-one-cultivar-out validation revealed substantial variation in predictive performance across held-out cultivars, with R^2^ and RPD ranging from 0.282 to 0.736 and from 1.180 to 1.945, respectively, for albumin, and from 0.416 to 0.750 and from 1.308 to 2.001, respectively, for globulin. Thus, although albumin achieved quantitative-level performance under the random train-test split, this performance was not consistently maintained for unseen cultivars, whereas globulin was generally more suitable for screening. SHapley additive explanations (SHAP) and partial dependence plots (PDPs) revealed that visible wavelengths contributed strongly to all models, with near-infrared (NIR) wavelengths supplying complementary information for globulin and the metabolic-protein sum. Overall, this framework offers rapid, interpretable flour-based estimation of metabolically active wheat protein fractions.

## 1. Introduction

Wheat (*Triticum aestivum* L.) is a major dietary protein source [1]. According to Osborne fractionation, grain proteins are classified as water-soluble albumins, salt-soluble globulins, alcohol-soluble gliadins, and alkali-soluble glutenins [2]. Albumins and globulins are commonly regarded as metabolically active protein fractions. They are rich in enzymes and polypeptide inhibitors and participate extensively in grain development, primary metabolism, and responses to environmental stress. In this study, metabolic protein denotes a derived sum calculated as albumin plus globulin; it is therefore not an independent third measurement. The two measured fractions and their derived sum can provide an integrated indicator of nutrition-related metabolically active proteins in wheat grain [3]. Their abundance varies with genotype, water supply, nitrogen, and fertilization [4,5], creating the phenotypic gradients needed for spectral prediction.

Wheat protein fractions are conventionally quantified by Osborne sequential extraction combined with colorimetric, chromatographic, or related chemical methods [2,6]. Although reliable, these methods are laborious and poorly suited to high-throughput phenotyping. Hyperspectral imaging (HSI), which integrates spectral and spatial information, has been widely applied to predicting wheat protein content, moisture, hardness, and other quality traits [7,8,9]. However, quantitative spectral prediction of specific protein fractions remains challenging. First, the N-H, C-H, and O-H absorption features of different protein fractions overlap, limiting their spectral discrimination [10]. Second, flour spectra also reflect variation in starch, moisture, lipids, pigments, particle size, and scattering, meaning that protein-related signals are embedded within a complex optical matrix [11].

Spectral preprocessing is important for enhancing informative variation and predictive performance [12]. Fractional-order derivative (FOD) transformation can reduce spectral overlap and emphasize subtle local features obscured in raw spectra [13]. FOD has shown potential for predicting soil organic carbon, crop physiological parameters, and agricultural-product quality [14,15]. Nevertheless, the order-dependent response patterns, informative wavelengths, and predictive performance of derivative-transformed spectra have not been systematically evaluated for albumin and globulin contents and the metabolic-protein sum.

Selecting informative wavelengths can reduce spectral redundancy while improving model parsimony and accuracy [16]. Uninformative variable elimination (UVE), competitive adaptive reweighted sampling (CARS), and the successive projections algorithm (SPA) reduce dimensionality by removing unstable variables, adaptively retaining informative variables, and minimizing collinearity, respectively [17]. Minimum redundancy maximum relevance (MRMR) ranking combined with recursive feature elimination (RFE) instead maximizes target relevance while reducing redundancy [18]. However, their relative effectiveness for predicting individual wheat protein fractions remains unclear.

Partial least squares regression (PLSR), support vector regression (SVR), and random forest (RF) are widely used in agricultural spectral analysis [19]. However, small-sample, high-dimensional spectral datasets can challenge conventional models because of variable redundancy and sensitivity to model configuration. Tabular prior-data fitted network (TabPFN) is a Transformer-based prior model pretrained on tabular tasks that enables rapid inference on small datasets with limited task-specific tuning [20]. Its potential for spectral prediction of wheat protein fractions, particularly with derivative transformation, remains to be explored. SHapley additive explanations (SHAP) quantify wavelength contributions from global and local perspectives [21], whereas partial dependence plots (PDPs) describe the marginal dependence of predictions on selected variables [22]. Together, they can identify influential wavelengths and clarify model-level spectral responses.

Based on these considerations, this study used whole-wheat flour samples from two complementary field experiments to evaluate the predictability of albumin and globulin contents and their derived sum, referred to here as the metabolic-protein sum (albumin + globulin), using visible and near-infrared (Vis-NIR) hyperspectral information. The objectives were to: (1) quantify variation in albumin and globulin contents and their metabolic-protein sum under water-nitrogen and water-fertilizer treatments; (2) identify target-specific optimal derivative orders across candidate orders from 0.0 to 2.0; (3) compare TabPFN with PLSR, SVR, and RF under the same data partition; (4) compare the accuracy-dimension trade-offs of MRMR-RFE, UVE, CARS, and SPA; (5) characterize model-level wavelength contributions using SHAP and PDP analyses; (6) assess analytical reproducibility through independent resampling validation and predictive stability through bootstrap resampling of the training context, with the derivative orders and wavelength subsets held fixed in both analyses; and (7) evaluate the heterogeneity of cross-cultivar transferability using leave-one-cultivar-out validation. This study aimed to establish an interpretable flour-based spectral phenotyping framework for estimating metabolically active wheat protein fractions.

## 2. Materials and Methods

### 2.1. Experimental Design and Sample Preparation

To construct a winter-wheat sample population with a broad gradient in protein composition, two complementary field experiments were conducted in spatially separate fields during the 2024–2025 growing season at the Dryland Water-Saving Agricultural Experimental Station of the Hebei Academy of Agriculture and Forestry Sciences, Shenzhou, Hebei Province, China. The station is located on the North China Plain and has a warm-temperate semi-arid monsoon climate, with a mean annual temperature of 13.4 °C and mean annual precipitation of approximately 480.7 mm. The soil is a deep sandy loam with moderate organic-matter content and is representative of the regional winter wheat-summer maize rotation system.

Experiment 1: Multi-cultivar water-nitrogen trial

Five winter wheat cultivars, namely J2, H2, H3, H6, and H7, were grown under selected combinations of irrigation regimes and nitrogen application rates. The irrigation regimes included 0S, rain-fed, no irrigation; 1S, moderate irrigation with 1050 m^3^ ha^−1^ applied at jointing; and 2S, sufficient irrigation with 1050 m^3^ ha^−1^ applied at both jointing and grain filling, totaling 2100 m^3^ ha^−1^. Nitrogen rates, expressed as elemental N, included 0N, zero nitrogen; 8N, moderate nitrogen with 105 kg N ha^−1^ as basal fertilizer and 155 kg N ha^−1^ as topdressing at jointing; and 16N, high nitrogen with 210 kg N ha^−1^ as basal fertilizer and 310 kg N ha^−1^ as topdressing at jointing.

Among the possible water-nitrogen combinations, five treatments were selected: 0S16N, 1S16N, 2S16N, 2S8N, and 2S0N. This experiment was not designed as a complete factorial trial. Therefore, treatment comparisons focused on two aspects: the effect of irrigation regime under high nitrogen supply, namely 0S16N, 1S16N, and 2S16N; and the effect of nitrogen rate under sufficient irrigation, namely 2S16N, 2S8N, and 2S0N. Each treatment was replicated six times in plots measuring 6 m × 3 m.

Experiment 2: Single-cultivar water-fertilizer full factorial trial

Cultivar H1 was subjected to a 4 × 4 factorial combination of four irrigation levels and four fertilization modes. The irrigation levels were W0, rain-fed with no irrigation; W1, moderate irrigation with 1200 m^3^ ha^−1^ applied at jointing; W2, moderate irrigation with 1200 m^3^ ha^−1^ applied at both jointing and booting, totaling 2400 m^3^ ha^−1^; and W3, sufficient irrigation with 1200 m^3^ ha^−1^ applied at jointing, booting, and grain filling, totaling 3600 m^3^ ha^−1^. The fertilization modes were C0, no fertilizer; C1, inorganic fertilizer only, with 220 kg N ha^−1^ as basal fertilizer and 330 kg N ha^−1^ as topdressing; C2, organic-inorganic combined fertilization, with 110 kg N ha^−1^ as basal fertilizer plus 30 t ha^−1^ composted manure and 165 kg N ha^−1^ as topdressing; and C3, organic fertilizer only, with 60 t ha^−1^ composted manure and no inorganic fertilizer. The experiment consisted of 16 treatment combinations, each with three replications in plots measuring 16.5 m × 10 m.

**Figure 1:**
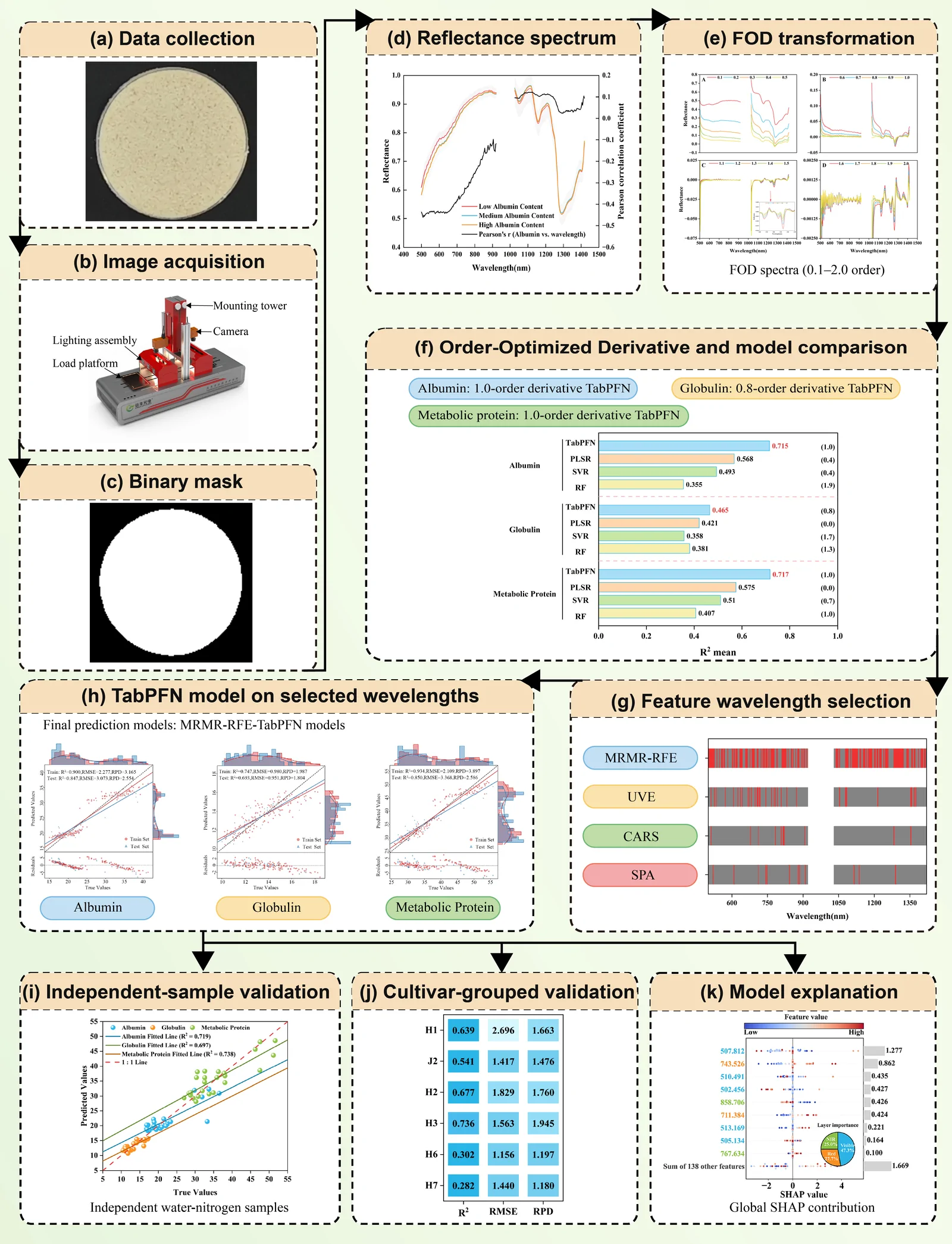
Workflow for sample preparation, hyperspectral preprocessing, derivative-order screening, wavelength selection, model development, independent re-sampling validation, cultivar-grouped validation, and model interpretation.

At maturity, grain was harvested from each plot, threshed, air-dried naturally, and ground to pass a 100-mesh sieve. The resulting flour was used for protein extraction and spectral acquisition (Figure 1). The sample preparation procedure was identical for the two experiments.

### 2.2. Extraction and Quantification of Protein Fractions

Albumin and globulin were sequentially extracted from whole-wheat flour using a modified Osborne procedure [2]. Briefly, 0.5 g of whole-wheat flour was weighed into a 50 mL centrifuge tube and mixed with 20 mL of distilled water. The suspension was shaken at 220 r min^−1^ for 2 h at 25 °C in a thermostatic shaker, and then centrifuged at 10,000 r min^−1^ for 15 min at 4 °C. The supernatant was collected, and the residue was re-extracted twice with 15 mL of distilled water under the same conditions. All supernatants were combined and made up to 100 mL with distilled water to obtain the albumin extract. The residue was then extracted analogously with 20 mL 10% (*w*/*v*) NaCl followed by two 15 mL extractions; pooled supernatants were diluted to 100 mL with 10% NaCl to obtain the globulin extract. Both extracts were stored at 4 °C until analysis.

Protein concentrations were determined using the Bradford method [23]. For each independent flour sample, the obtained albumin or globulin extract was divided into three aliquots. From each aliquot, a 1.0 mL portion was mixed with 5.0 mL of Coomassie Brilliant Blue G-250 working solution. After thorough mixing, the reaction was allowed to proceed at room temperature for 2 min, and the absorbance was measured three times at 595 nm using a SpectraMax iD3 multimode microplate reader (Molecular Devices, San Jose, CA, USA). Bovine serum albumin (BSA) was used as the standard. Standard solutions were prepared in the solvent corresponding to each extract, namely distilled water for albumin and 10% NaCl for globulin. Albumin and globulin contents were calculated from the respective standard curves. The metabolic-protein sum was not measured independently; it was calculated for each sample by adding the measured albumin and globulin contents.

The final protein concentration for each independent flour sample was calculated as the mean of the aliquot-level results obtained from repeated measurements. Across the albumin and globulin measurements, the coefficients of variation for replicate aliquots prepared from the same protein extract ranged from 6.68% to 9.47%, whereas those for repeated instrumental measurements of the same aliquot ranged from 3.58% to 5.12%. The variability associated with independent replicate extractions and its contribution to the overall measurement uncertainty were not assessed in this study. The moisture content of the whole-wheat flour samples ranged from 11.46% to 13.28%. Protein-fraction contents were calculated and reported on an as-is flour basis, without conversion to a dry-matter basis, and are expressed as mg g^−1^ of the analyzed flour.

### 2.3. Hyperspectral Image Acquisition

Hyperspectral images of whole-wheat flour were acquired using a RAP-HHIS-Q hyperspectral quality analyzer (GreenPhone, Shanghai, China) equipped with a halogen light source and a dual-camera line-scan imaging system. The Vis-NIR camera covered the 400–1000 nm spectral range, with a spectral resolution of 2.8 nm and 1312 spatial pixels per line. The short-wave infrared (SWIR) camera covered the 900–1700 nm spectral range, with a spectral resolution of 6.0 nm and 320 spatial pixels per line. Images were acquired in a light-tight enclosure. Each flour sample was placed in a transparent dish on a matte-black tray, and the translation-stage speed was 15 mm s^−1^.

The acquired hyperspectral cubes were converted to reflectance using dark (lens covered) and white (99% diffuse reflectance standard) references. After excluding uninformative regions at both spectral ends, two regions, 499.78–920.31 and 1025.89–1418.75 nm, were retained, comprising 268 effective bands for subsequent analysis.

### 2.4. Spectral Feature Extraction and Preprocessing

Each hyperspectral image contained both the whole-wheat flour sample and the surrounding background. A threshold-based segmentation algorithm integrated into the instrument software was used to generate a binary mask, removing background pixels and retaining valid sample pixels as the region of interest (ROI). A representative spectrum for each sample was then obtained by averaging the spectra of all ROI pixels [24].

After extraction of the spectral data, FOD was employed for data preprocessing. FOD is a generalized form of integer-order differentiation, extending conventional first- and second-order derivatives to non-integer orders. In this study, the G-L formulation was adopted [25]:

(1) $$D^{v}f(t)=\lim_{h\to 0}\frac{1}{h^{v}}\sum_{i=0}^{\left\lfloor (t-a)/h\right\rfloor }(-1{)}^{i}\frac{\Gamma (v+1)}{i!\Gamma (v-i+1)}f(t-ih)$$

where v is the fractional-order, h is the step size, t and a are the upper and lower limits of the differentiation, respectively, and Γ is the Gamma function as follows:

(2) $$\Gamma (\tau )=\int_{0}^{\infty }e^{-u}u^{\tau -1}\mathrm{du}$$

For discrete spectral data, the G-L derivative can be approximated as:

(3) $$\frac{d^{v}f(t)}{dt^{v}}\approx f(t)-vf(t-1)+\frac{v(v-1)}{2}f(t-2)+\cdots +(-1{)}^{i}\frac{\Gamma (v+1)}{i!\Gamma (v-i+1)}f(t-i)$$

In this study, the FOD order v ranged from 0.0 to 2.0 in increments of 0.1. The two retained spectral segments (499.78–920.31 and 1025.89–1418.75 nm) were processed independently on their native wavelength grids and concatenated after transformation; no wavelength interpolation was performed. For each segment, h was set to the actual interval between adjacent wavelengths. G-L weights were calculated recursively, and the full preceding sequence within each segment was used at each wavelength. At v = 0, the transformed spectrum was identical to the original spectrum; at integer values of v, the procedure corresponded to conventional integer-order differentiation. Because the G-L method calculates derivative spectra using a finite-difference strategy, the number of spectral bands after transformation was reduced relative to the original spectral matrix.

Here, the order-optimized derivative preprocessing (ODP) method used FOD to transform the raw spectra and systematically screened the candidate derivative orders through cross-validation performed exclusively on the training set. This procedure identified the target-specific optimal derivative order for each protein indicator.

### 2.5. Dimensionality Reduction Technique

Following spectral preprocessing, four wavelength selection algorithms were compared to identify informative variables for predicting metabolically active protein fractions. ODP was applied separately to each target to obtain the corresponding optimal derivative order. The spectra transformed using the optimal derivative order for each target were subsequently used as inputs for UVE, CARS, SPA, and MRMR-RFE.

The UVE algorithm removes variables with stability lower than that of artificial noise variables in a PLSR framework [26]. The CARS algorithm combines Monte Carlo sampling, exponentially decreasing functions, and adaptive reweighted sampling to retain variables with larger regression contributions [27]. The SPA iteratively selects wavelengths with low collinearity through orthogonal projection [28]. The MRMR-RFE procedure first generated an ordered ranking of spectral variables using the MRMR criterion, which maximized target relevance while minimizing redundancy. Nested feature subsets were then constructed according to this fixed ranking by progressively excluding the lowest-ranked variables. For each target, the maximum mean cross-validation R^2^ among all candidate subsets was first identified. Subsets with a mean cross-validation R^2^ greater than or equal to 98% of this maximum value were considered near-optimal candidates, and the subset containing the smallest number of wavelengths among these candidates was retained as the final MRMR-RFE feature set.

### 2.6. Development of Regression Models

Albumin and globulin contents and the metabolic-protein sum were predicted using both full-spectrum variables and selected wavelengths. Four regression algorithms were compared: PLSR, SVR, RF, and TabPFN. All models were implemented in Python 3.10.18 using scikit-learn 1.6.1, PyTorch 2.5.1, and the TabPFN 6.4.1. The random seed was fixed at 42 for data splitting and all stochastic modeling and re-sampling procedures.

For PLSR, the number of latent variables was optimized from 1 to 20. SVR used a radial-basis-function kernel, with C searched from 10^−3^ to 10^3^ and γ searched from 10^−4^ to 10^0^. For RF, the tuned hyperparameters were the number of trees (100–800), maximum depth (3–20), minimum samples per split (2–10), and minimum samples per leaf (1–5). Optimal hyperparameters for PLSR, SVR, and RF were selected by Bayesian optimization with five-fold cross-validation on the training set. Mean cross-validation performance was used as the optimization objective.

TabPFN is a Transformer-based model pretrained on synthetic tabular tasks and can generate predictions with minimal task-specific tuning [20]. The regression variant of TabPFN was used with its default architecture. To ensure comparability, all models used the same training and test partitions.

### 2.7. Model Evaluation and Validation

The dataset comprised 198 wheat flour samples, which were randomly split into a training set of 158 samples (80%) and a test set of 40 samples (20%). All preprocessing, derivative-order selection, wavelength selection, and hyperparameter tuning were performed exclusively within the training set using five-fold cross-validation. The test set remained completely unseen during model development and was used only for final evaluation.

Model performance was evaluated using the coefficient of determination (R^2^), root mean square error (RMSE), and residual prediction deviation (RPD). R^2^ is a goodness-of-fit metric that quantifies the proportion of variance in the reference values explained by the predicted values. RMSE measures the average magnitude of prediction error, with lower values indicating better performance. RPD was calculated as the standard deviation of the reference values divided by RMSE, with higher values indicating stronger predictive capability.

### 2.8. Independent Re-Sampling Validation and Bootstrap Stability Assessment

For independent re-sampling validation, 25 composite samples (five cultivars × five water-nitrogen treatments) were prepared by re-sampling grain material from the corresponding Experiment 1 harvests. These samples were independently ground, scanned, and chemically quantified using the same procedures and were excluded from the 198-sample dataset and all model-development steps. Because the material originated from the same field harvest, this procedure assesses analytical reproducibility rather than external biological generalization. For the metabolic-protein sum, the directly trained model was compared with a derived summed prediction obtained by adding the independently predicted albumin and globulin contents. The fitted-line R^2^ values from the linear regression were used only to illustrate the association between the measured and predicted values. Predictive accuracy was evaluated directly using the measured values and the model predictions, with R^2^, RMSE, and mean bias (MB) as the evaluation metrics. MB was calculated as the average difference between the predicted and measured values; positive values indicate systematic overprediction, whereas negative values indicate systematic underprediction.

To assess the sensitivity of the optimized models to variation in the training-data context, a bootstrap-based stability analysis was conducted. The target-specific derivative orders and MRMR-RFE wavelength subsets determined during the original model-development procedure were kept fixed. At each bootstrap iteration, 158 observations were sampled with replacement from the original 158-sample training set and used as the context data for the standard TabPFN regression model. The same fixed 40-sample holdout set was used for evaluation. The procedure was repeated 100, 200, and 400 times using random seed 42. For each target and repetition number, the distribution of test-set R^2^ values was summarized by its median and empirical 95% percentile interval, calculated as the 2.5th and 97.5th percentiles of the bootstrap R^2^ distribution. These intervals therefore quantify the variability in test-set R^2^ induced by bootstrap resampling of the training context, conditional on the fixed derivative order, fixed wavelength subset, and fixed test set.

### 2.9. Model Interpretability Analysis

To elucidate model response patterns for albumin, globulin, and the metabolic-protein sum, post hoc interpretability analyses were conducted using SHAP. KernelExplainer was used to quantify wavelength contributions, and global importance was summarized using the mean absolute SHAP value. For spectral-region analysis, retained wavelengths were assigned to three nonoverlapping operational regions: visible (499.78–599.99 nm), red (600.00–749.99 nm), and NIR (750.00–920.31 and 1025.89–1418.75 nm); the unmeasured gap between the two camera segments was excluded. Regional contributions were calculated by summing mean absolute SHAP values within each region and normalizing by the total across all retained features.

PDPs and individual conditional expectation (ICE) curves were used to describe marginal predictive dependence and sample-level heterogeneity at key wavelengths [29]. The resulting curves were interpreted in conjunction with known optical scattering effects and NIR vibration assignments. These analyses describe model-level associations and do not establish direct molecular causation.

### 2.10. Cultivar-Grouped Validation

A leave-one-cultivar-out grouped validation strategy was used to assess the cross-cultivar applicability of the ODP-MRMR-RFE-TabPFN framework. To evaluate the ability of the optimized models to predict samples from cultivars not represented in the training data, the six cultivars included in the experiments, namely H1, J2, H2, H3, H6, and H7, were treated as six independent cultivar groups. In each validation round, all samples belonging to one cultivar, including samples collected under different irrigation, nitrogen, or fertilizer treatments, were assigned to the test set, while samples from the other five cultivars were used as the training set. Within each training set, cross-validation was used to determine the optimal derivative order and wavelength subset. The optimized ODP-MRMR-RFE-TabPFN models for albumin and globulin contents and the metabolic-protein sum were then evaluated separately on the corresponding held-out cultivar group. For each held-out cultivar group, model performance was evaluated using the R^2^, RMSE, and RPD.

### 2.11. Statistical Analysis of Treatment Effects

Treatment differences in protein-fraction contents were analyzed using IBM SPSS Statistics 27.0. For Experiment 1, one-way analysis of variance (ANOVA) was conducted separately within each cultivar, with the five water-nitrogen treatments treated as independent levels and six biological replicates per treatment. For Experiment 2, irrigation level and fertilization mode were treated as fixed factors in a two-way ANOVA. Each water-fertilizer combination included three biological replicates. Significant ANOVAs were followed by Duncan’s multiple range test at *p* < 0.05.

## 3. Results

### 3.1. Variation in Grain Protein Fractions Under Water-Nitrogen and Water-Fertilizer Treatments

**Figure 2:**
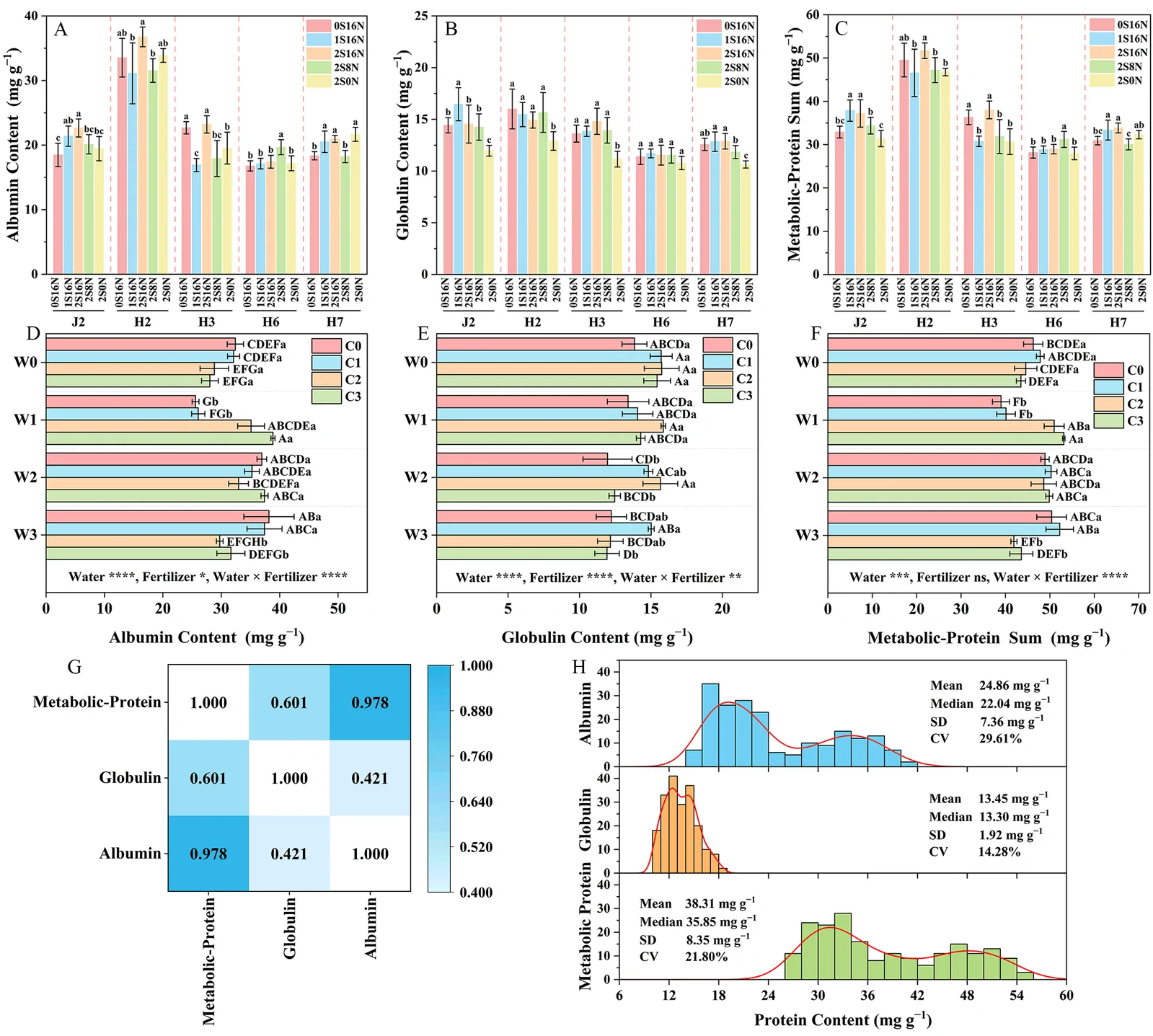
Protein-fraction contents under water-nitrogen and water-fertilizer treatments. (**A**–**C**) Five cultivars under five water-nitrogen treatments. (**D**–**F**) H1 under 16 water-fertilizer combinations. (**G**) Pearson correlation analysis. (**H**) Frequency distributions and descriptive statistics. In panels (**A**–**C**), bars are means ± SD (*n* = 6), and different lowercase letters compare treatments within each cultivar. In panels (**D**–**F**), bars are means ± SD (*n* = 3); different uppercase letters indicate differences among treatment combinations, whereas different lowercase letters indicate differences within the corresponding groups. Significance levels are indicated as follows: **** *p* < 0.0001, *** *p* < 0.001, ** *p* < 0.01, * *p* < 0.05, and ns, not significant. Letters were assigned using Duncan’s multiple range test at *p* < 0.05. Protein contents are expressed as mg g^−1^.

Variation in grain protein-fraction contents among wheat cultivars under the five water-nitrogen treatments is shown in Figure 2A–C. Cultivar H2 maintained relatively high protein-fraction levels across treatments, whereas H6 generally showed lower levels. Under high nitrogen supply (16N), increasing the number of irrigation events generally increased albumin content. Globulin content showed no significant response to the irrigation gradient in most cultivars, except for J2. Under sufficient irrigation (2S), protein fraction contents generally decreased with decreasing nitrogen application rate, except in H6, where changes were comparatively small.

Figure 2D–F presents the variation in protein fraction contents of H1 wheat grains under different water-fertilizer combinations. Two-way analysis of variance showed that irrigation, fertilization, and their interaction significantly affected both albumin and globulin contents. Albumin content remained relatively high under the W1C3, W2C3, W3C0, and W3C1 treatments. Globulin content showed a relatively narrow range of variation overall, with comparatively higher values observed under W0C1, W0C2, and W1C2. The metabolic-protein sum was mainly affected by irrigation and the irrigation × fertilization interaction. Compared with the other treatment combinations, its content was significantly higher under W1C2, W1C3, W2C0, W2C1, W2C2, W2C3, W3C1, and W3C0, indicating that moderate irrigation, particularly the W2 treatment, favored its accumulation.

The overall variation in grain protein fractions across cultivars and treatment combinations is summarized in Figure 2H. The mean albumin content was 24.86 mg g^−1^, with a standard deviation of 7.36 mg g^−1^ and a coefficient of variation of 29.61%, whereas the mean globulin content was 13.45 mg g^−1^, with a standard deviation of 1.92 mg g^−1^ and a coefficient of variation of 14.28%. Albumin content exhibited moderate to high variability, whereas globulin content showed relatively low variability. The metabolic-protein sum was strongly correlated with albumin content (Figure 2G) and showed a similarly high level of variability.

### 3.2. Spectral Responses and Correlation Patterns Associated with Protein Fraction Contents

**Figure 3:**
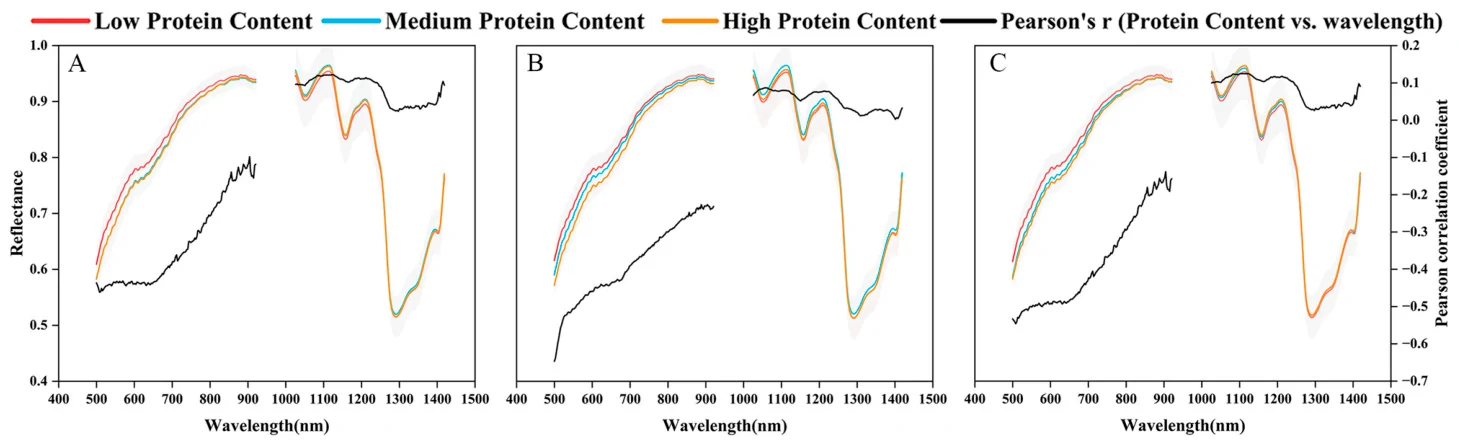
Raw Vis-NIR spectra of whole-wheat flour samples and wavelength-wise Pearson correlations with (**A**) albumin, (**B**) globulin, and (**C**) the metabolic-protein sum. The shaded area indicates the range of the raw spectra.

The mean spectra of whole-wheat flour samples grouped by protein fraction content showed similar overall profiles (Figure 3). Reflectance gradually increased within the 500–950 nm range, exhibited local fluctuations around 1000–1150 nm, and showed absorption valleys in the 1200–1350 nm region. Group separations were most evident near 650, 1100, 1200, and 1350 nm. Pearson correlations were generally stronger in the visible and red regions than in the NIR, with the strongest raw-spectrum correlations mainly within 500–900 nm.

### 3.3. Effects of Fractional-Order Derivative Transformation on Spectral Features and Protein-Related Correlations

**Figure 4:**
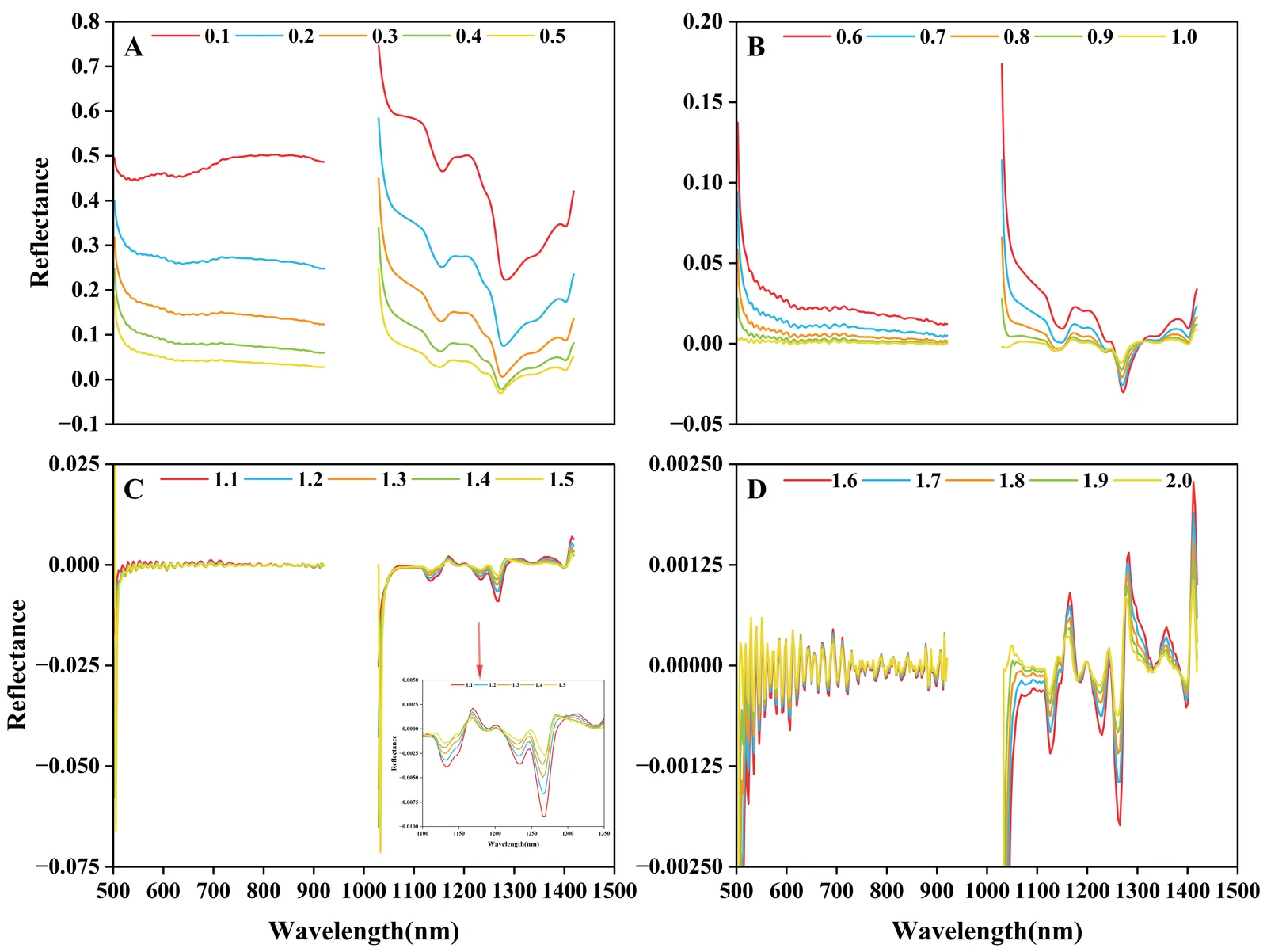
Average spectral curves of whole-wheat flour samples after fractional-order derivative transformation. (**A**–**D**) Mean spectral curves processed using FOD orders of 0.1–0.5, 0.6–1.0, 1.1–1.5, and 1.6–2.0, respectively. The inset in subplot (**C**) provides an enlarged view of local spectral variations.

The spectral curves of whole-wheat flour samples after FOD transformation are shown in Figure 4. As the FOD order increased, the number of spectral peaks increased markedly, with more pronounced features around 510, 1150, and 1300 nm, while the dynamic range of the transformed spectra gradually narrowed toward zero. However, when the FOD order exceeded 1.5, the spectral curves showed intensified fluctuations and a certain degree of distortion, suggesting that high-order FOD transformation may amplify noise information.

**Figure 5:**
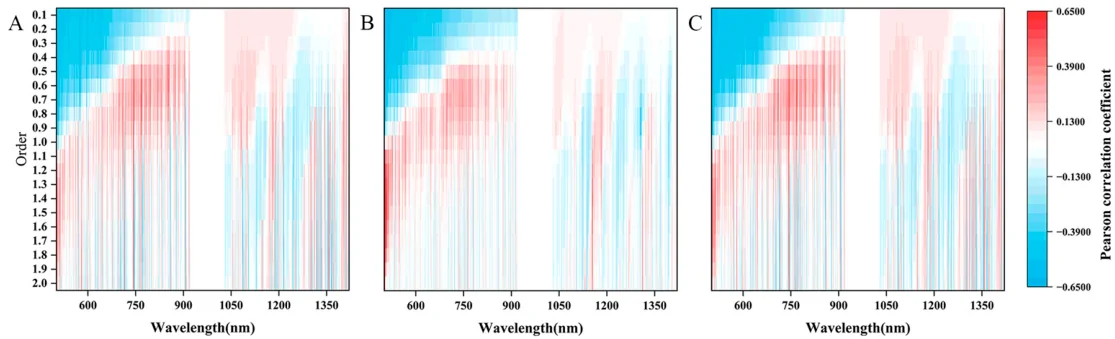
Pearson correlation heatmaps between protein fraction contents and FOD-transformed Vis-NIR spectra across different derivative orders and wavelengths. (**A**) Albumin, (**B**) globulin, and (**C**) the metabolic-protein sum.

The distributions of Pearson correlation coefficients between the target protein contents and FOD-transformed spectra across derivative orders are shown in Figure 5. Overall, albumin and globulin contents and the metabolic-protein sum all showed strong and continuous correlation responses within the 0.5–1.0 order range, whereas the correlations generally weakened and became more scattered when the FOD order exceeded 1.5. The 500–900 nm region was the main sensitive spectral range for all targets, and the correlation patterns showed clear positive-to-negative transitions with increasing FOD order. Albumin and the metabolic-protein sum also exhibited distinct local responses around 1150, 1300, and 1350 nm.

### 3.4. Effects of FOD Order on Prediction Performance

**Figure 6:**
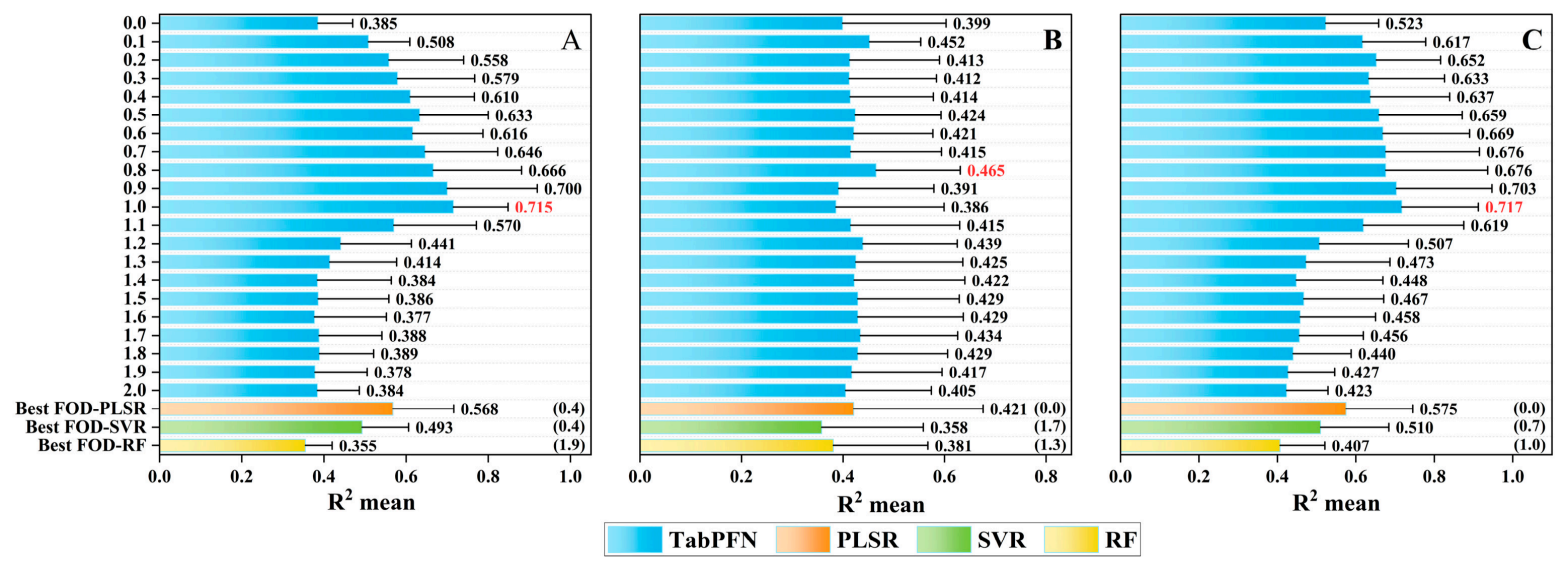
Comparison of mean five-fold cross-validation performance for protein fraction contents across different derivative orders and models. (**A**) Albumin, (**B**) globulin, and (**C**) the metabolic-protein sum. TabPFN is shown for orders 0.0–2.0; PLSR, SVR, and RF are shown at their optimal orders (in parentheses).

Figure 6 compares the prediction performance of the TabPFN model for albumin and globulin contents and the metabolic-protein sum using derivative-transformed spectra with orders ranging from 0.0 to 2.0. The cross-validation analysis identified target-specific optimal derivative orders. For albumin content, the TabPFN model achieved the best performance using order 1.0, which corresponds to the conventional first derivative, with a mean validation R^2^ of 0.715, RMSE of 3.717 mg g^−1^, and RPD of 1.981. For globulin content, the highest performance was obtained using order 0.8, a non-integer derivative, with a mean validation R^2^ of 0.465, RMSE of 1.357 mg g^−1^, and RPD of 1.405. For the metabolic-protein sum, the TabPFN model performed best using the 1.0-order derivative-transformed spectra, with a mean validation R^2^ of 0.717, RMSE of 4.119 mg g^−1^, and RPD of 2.083. Compared with PLSR, SVR, and RF using their respective derivative orders selected by ODP, TabPFN consistently achieved higher mean cross-validation accuracy across all targets.

TabPFN performance revealed clear order-dependent patterns for albumin, globulin, and the metabolic-protein sum (Figure 6). As the FOD order increased, prediction accuracy initially improved and reached its optimum around the 0.8–1.0 order range, after which model performance declined. When the derivative order increased to 2.0, the mean cross-validation R^2^ values for albumin, globulin, and the metabolic-protein sum decreased to 0.384, 0.405, and 0.423, respectively, while the corresponding RMSE values increased to 5.586, 1.382, and 6.153 mg g^−1^.

### 3.5. Feature Wavelength Selection and Validation of TabPFN Models

**Figure 7:**
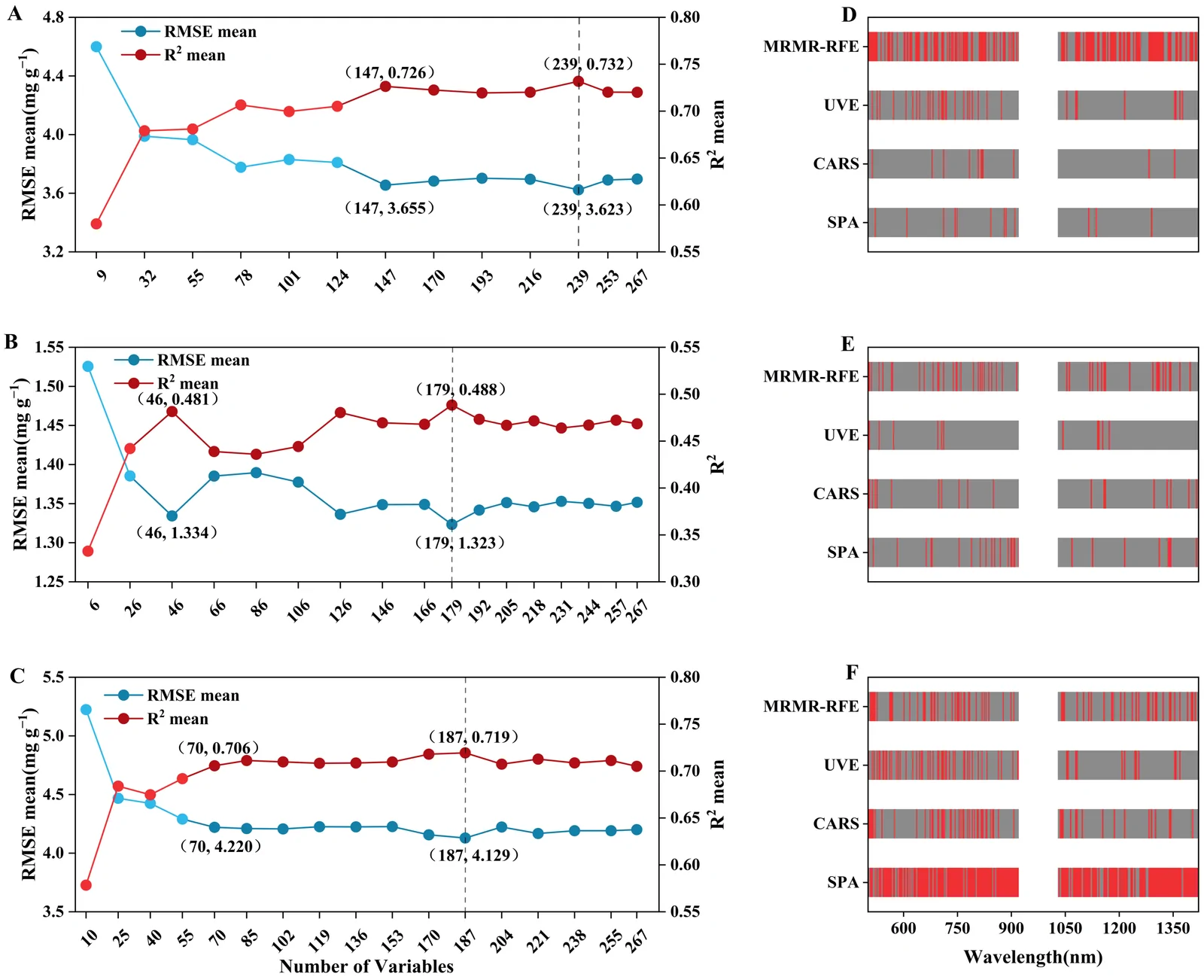
MRMR-RFE feature selection process and wavelength distributions. (**A**–**C**) Mean cross-validation RMSE and R^2^ values during MRMR-RFE feature selection for albumin, globulin, and the metabolic-protein sum, respectively. The color transition points indicate the feature numbers after which model performance tended to stabilize, and the vertical dashed lines mark the subsets with the highest predictive performance. (**D**–**F**) Wavelengths retained by MRMR-RFE, UVE, CARS, and SPA. Red bands indicate the retained spectral variables.

During the MRMR-RFE feature selection process, model prediction performance generally increased at the early stage and then gradually stabilized as the number of retained variables increased (Figure 7A–C). Considering both prediction accuracy and variable reduction, 147, 46, and 70 variables were selected as the final MRMR-RFE feature subsets for albumin, globulin, and metabolic protein contents, respectively. In comparison, UVE, CARS, and SPA retained 33, 11, and 12 feature wavelengths for albumin content; 12, 19, and 27 feature wavelengths for globulin content; and 59, 59, and 220 feature wavelengths for metabolic protein content, respectively. These differences indicate an inherent trade-off among different feature selection methods between removing redundant variables and preserving effective spectral information.

The effective spectral information associated with protein fraction prediction was distributed across multiple spectral regions rather than being confined to a single wavelength range (Figure 7D–F). Differences in the distribution of retained feature wavelengths further suggest that albumin, globulin, and the metabolic-protein sum exhibited distinct spectral response patterns.

**Figure 8:**
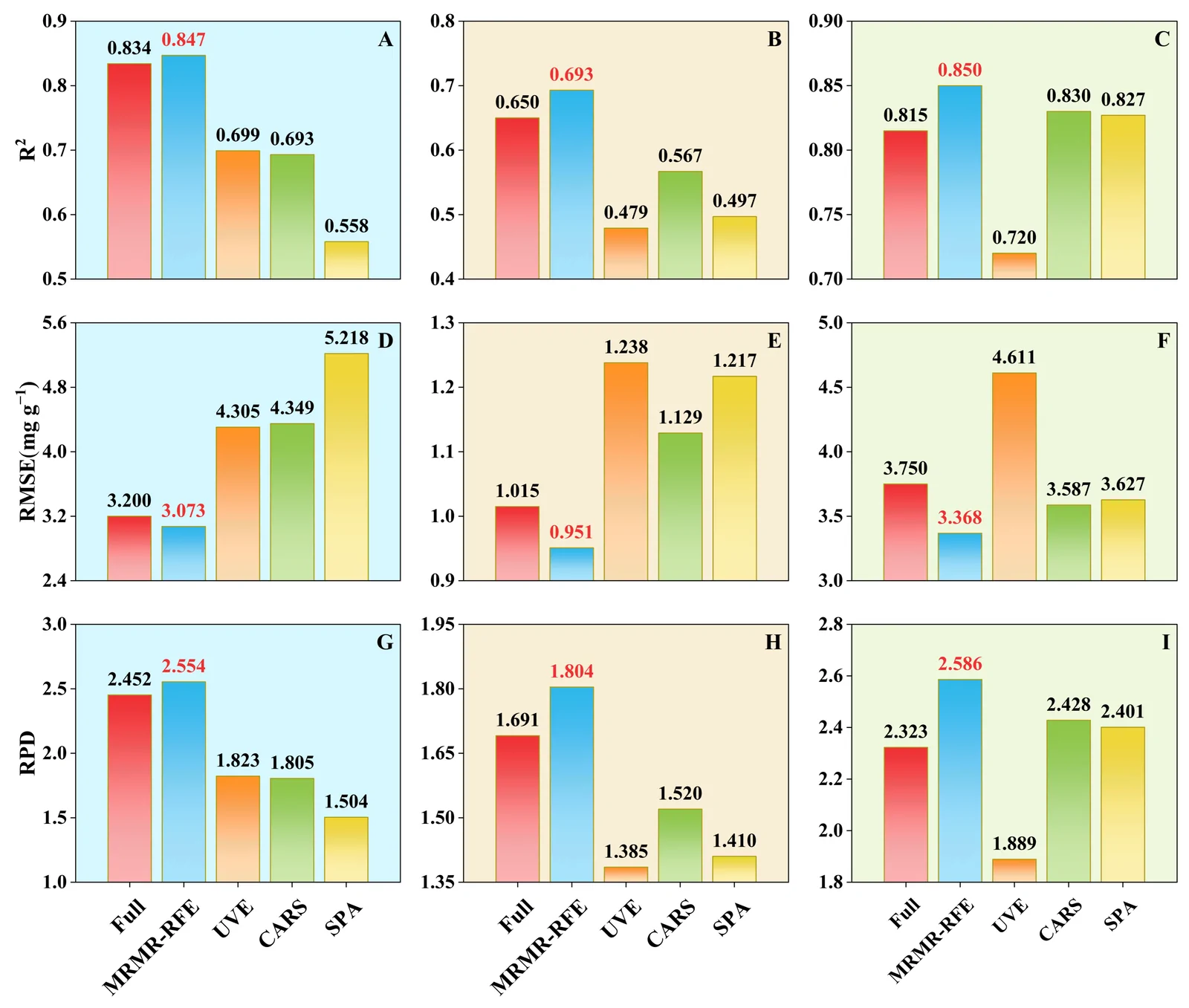
Evaluation of TabPFN prediction performance for albumin, globulin, and the metabolic-protein sum using full spectra and feature subsets. (**A**–**C**) R^2^, (**D**–**F**) RMSE, and (**G**–**I**) RPD. In each panel, bars represent the prediction performance of the full spectrum, MRMR-RFE, UVE, CARS, and SPA; the blue, orange, and green backgrounds correspond to albumin, globulin, and the metabolic-protein sum, respectively.

The prediction performance of TabPFN models using full-spectrum variables and different feature-selection strategies is shown in Figure 8 and Table A1. The relative performance of the feature-selection methods differed between cross-validation and test evaluation. MRMR-RFE achieved the highest mean cross-validation R^2^ for albumin, whereas CARS yielded slightly higher mean cross-validation R^2^, lower RMSE, and fewer retained wavelengths for globulin and the metabolic-protein sum. In contrast, on the test-set, the MRMR-RFE-based models achieved the highest R^2^ and RPD and the lowest RMSE among the evaluated feature-selection strategies for all three targets. Specifically, the albumin model obtained an R^2^ of 0.847, RMSE of 3.073 mg g^−1^, and RPD of 2.554; the globulin model obtained an R^2^ of 0.693, RMSE of 0.951 mg g^−1^, and RPD of 1.804; and the model for the metabolic-protein sum obtained an R^2^ of 0.850, RMSE of 3.368 mg g^−1^, and RPD of 2.586. These results indicate that the combined ODP-MRMR-RFE-TabPFN workflow provided strong predictive performance while reducing spectral dimensionality, although MRMR-RFE was not uniformly superior in cross-validation across all targets.

### 3.6. Independent Re-Sampling Validation of Protein-Fraction Prediction

**Figure 9:**
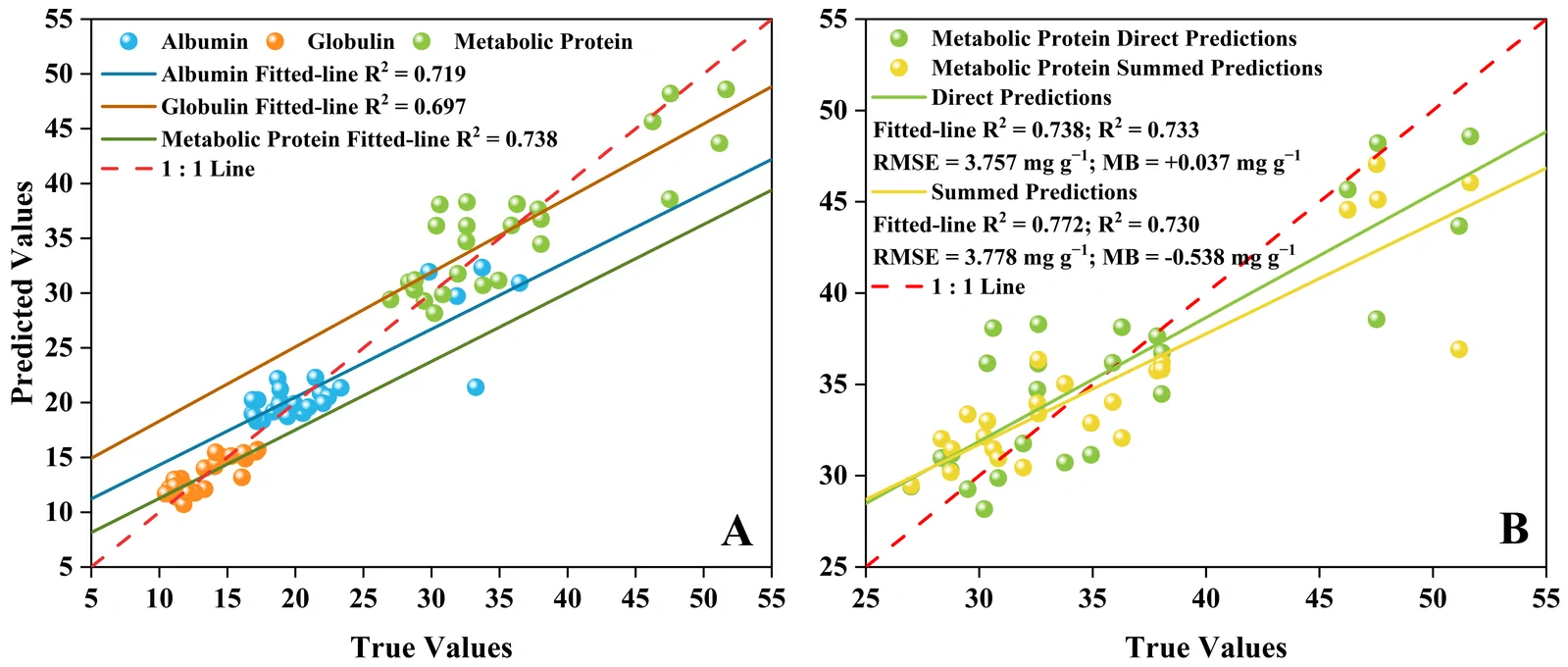
Comparison of measured and predicted protein-fraction contents in independent water-nitrogen samples using linear regression. (**A**) Albumin, globulin, and the metabolic-protein sum; (**B**) direct and component-summed predictions of the metabolic-protein sum. Fitted-line R^2^ values describe the linear association between measured and predicted values, whereas predictive R^2^, RMSE, and MB were calculated directly from the measured values and unchanged predictions to evaluate predictive agreement.

The results of the independent re-sampling validation are shown in Figure 9. The fitted-line R^2^ values for albumin, globulin, and the metabolic-protein sum were 0.719, 0.697, and 0.738, respectively (Figure 9A), indicating strong linear associations between measured and predicted values.

For the metabolic-protein sum, the direct and component-summed predictions showed comparable predictive performance (Figure 9B). The direct prediction yielded a predictive R^2^ of 0.733, an RMSE of 3.757 mg g^−1^, and an MB of +0.037 mg g^−1^, whereas the component-summed prediction yielded corresponding values of 0.730, 3.778 mg g^−1^, and −0.538 mg g^−1^. Thus, the two approaches produced nearly identical predictive R^2^ and RMSE values, although the component-summed prediction showed a greater negative bias. Overall, the direct prediction exhibited lower systematic bias in the independently re-sampled samples.

### 3.7. Bootstrap-Based Robustness Assessment of the Optimized Modeling Framework

**Figure 10:**
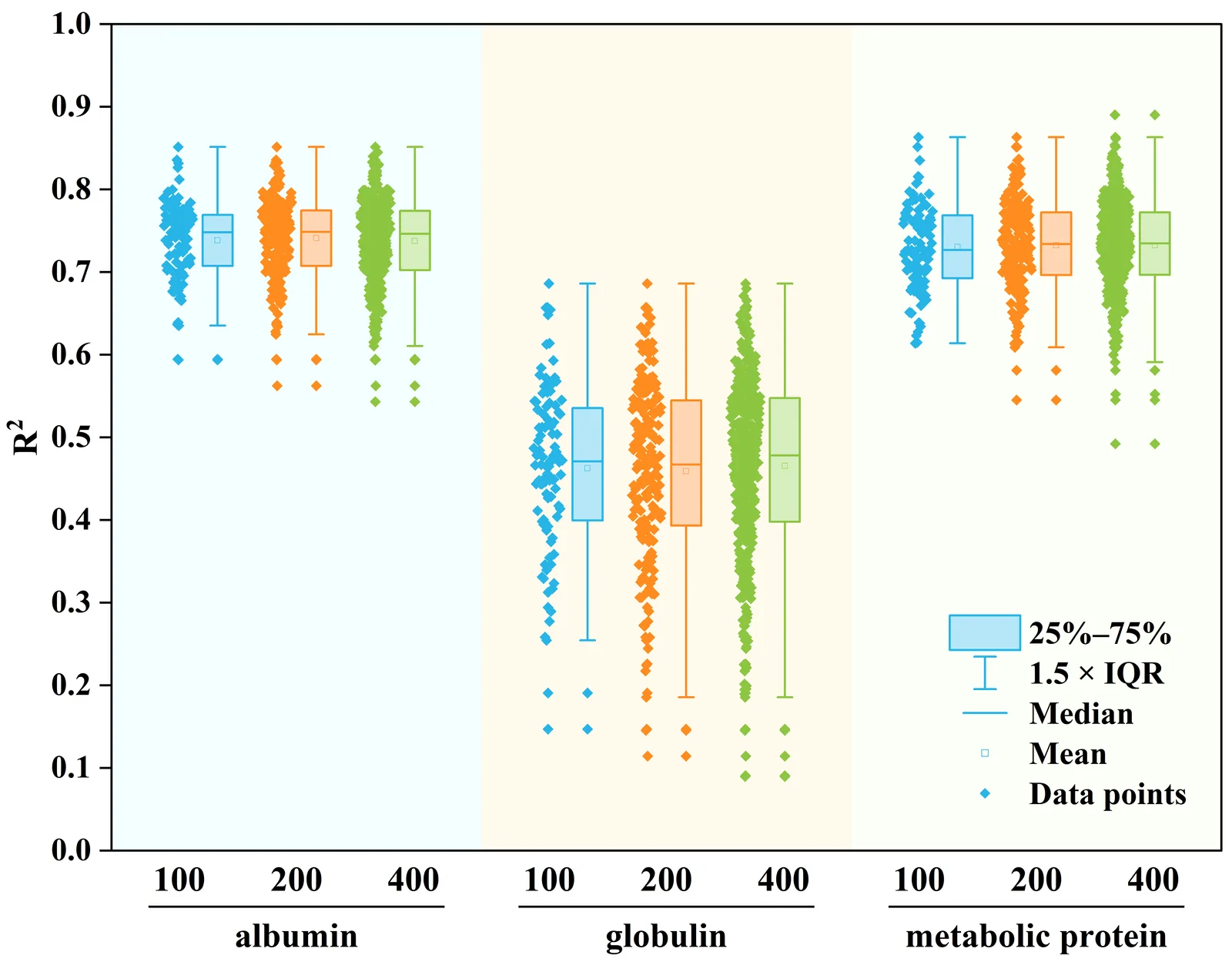
Bootstrap-based training-context resampling stability of the fixed-configuration ODP-MRMR-RFE-TabPFN models. Test-set R^2^ distributions are shown for albumin, globulin, and metabolic protein after 100, 200, and 400 bootstrap iterations.

The fixed-configuration TabPFN models showed generally stable test-set performance as the number of bootstrap repetitions increased, with only minor changes in the median R^2^ values (Figure 10). For albumin, the median test set R^2^ values after 100, 200, and 400 Bootstrap repetitions were 0.748, 0.749, and 0.749, respectively, with corresponding empirical 95% percentile intervals of 0.636–0.829, 0.634–0.829, and 0.634–0.829. For globulin, the median R^2^ values were 0.471, 0.478, and 0.478, respectively, with corresponding empirical 95% percentile intervals of 0.256–0.656, 0.199–0.649, and 0.199–0.649. The wider percentile intervals for globulin indicate greater sensitivity of its test-set R^2^ to variation in the bootstrap-resampled training context under the fixed modeling configuration. The bootstrap distribution of test-set R^2^ values for the metabolic-protein sum showed a trend similar to that of albumin, consistent with their strong Pearson correlation.

### 3.8. Model Interpretation Based on SHAP and PDP Analyses

To characterize predictive response patterns in the ODP-MRMR-RFE-TabPFN models, SHAP and PDP analyses were conducted using the target-specific optimal FOD order and MRMR-RFE feature subset. Albumin and the metabolic-protein sum were interpreted using 1.0-order derivative-transformed spectra, whereas globulin was interpreted using 0.8-order derivative-transformed spectra.

#### 3.8.1. SHAP-Based Contribution Analysis of Key Wavelengths and Spectral Regions

**Figure 11:**
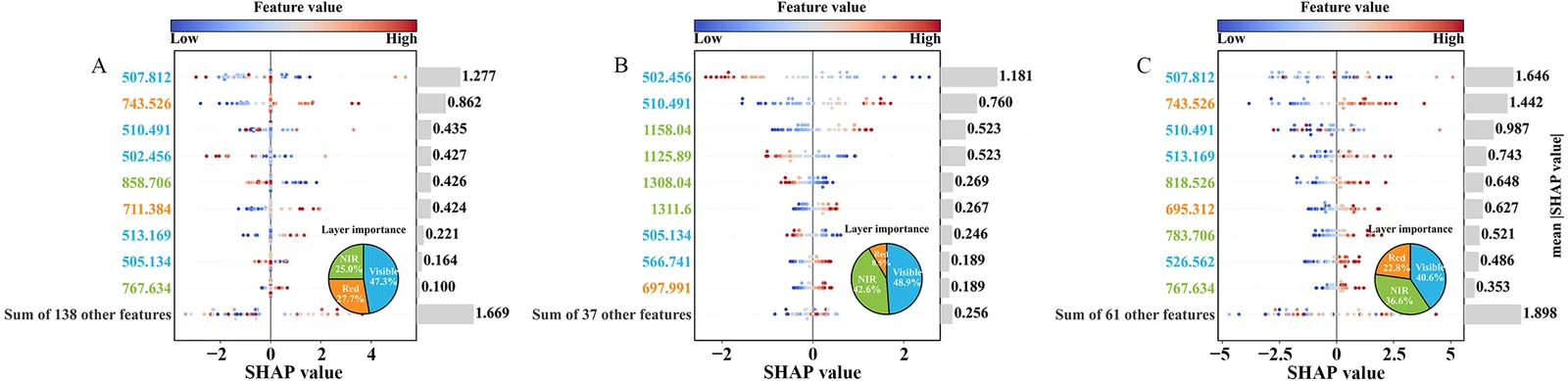
Global SHAP interpretation of ODP-MRMR-RFE-TabPFN models for (**A**) albumin, (**B**) globulin, and (**C**) the metabolic-protein sum. Beeswarm plots show the nine highest-importance wavelengths; inset charts summarize visible, red, and NIR contributions.

The global contributions of wavelength variables to the MRMR-RFE-TabPFN predictions of albumin, globulin, and metabolic protein are shown by SHAP beeswarm plots in Figure 11. For albumin content, 507.812 nm had the highest mean absolute SHAP value and was identified as the most influential wavelength, while other high-contribution wavelengths were mainly distributed in the 500–520 nm and 700–850 nm regions. For globulin content, 502.456 nm contributed the most to the model output, and important wavelengths were mainly concentrated in the 500–570 nm and 1120–1310 nm regions. For metabolic protein content, 507.812 nm was also the most important wavelength, with other high-contribution wavelengths mainly located in the 500–530 nm and 740–820 nm regions.

In terms of spectral-region contributions, the visible, red, and NIR regions contributed 47.3%, 27.7%, and 25.0% to the albumin model, respectively. For the globulin model, their contributions were 48.8%, 8.5%, and 42.7%, respectively. For the model predicting the metabolic-protein sum, the contributions were 40.6%, 22.8%, and 36.6%, respectively. Overall, the visible region contributed the most to all models, whereas the NIR region contributed more than 35% to both the globulin and metabolic-protein-sum models. These results indicate clear differences in the use of Vis-NIR spectral information among different protein fraction prediction models.

#### 3.8.2. SHAP Dependence and PDP Responses of Key Wavelengths

**Figure 12:**
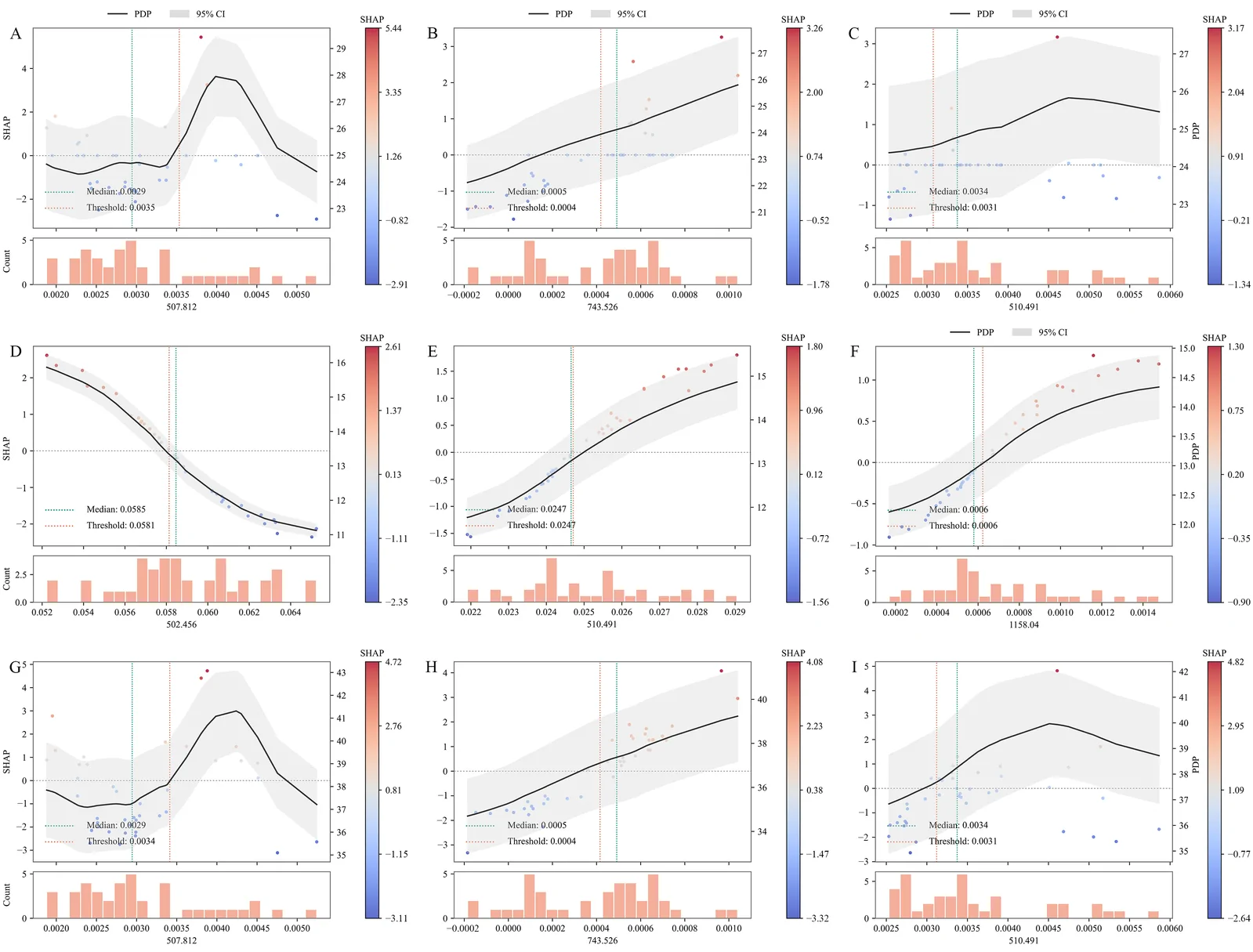
SHAP dependence and PDPs for the top three wavelengths in the models for (**A**–**C**) albumin, (**D**–**F**) globulin, and (**G**–**I**) the metabolic-protein sum. Points are sample SHAP values; black lines and gray bands show PDPs and 95% confidence intervals. Dotted lines mark median feature values and estimated thresholds; histograms show feature distributions. Each point represents one sample.

The SHAP dependence relationships and PDP responses of the three most important wavelengths in the albumin, globulin, and metabolic protein models are shown in Figure 12. For the albumin model, 507.812 and 510.491 nm both showed nonlinear threshold responses. PDP values increased rapidly when the feature value exceeded approximately 0.0030, reached a peak in the high feature-value range, and then declined. In contrast, 743.526 nm showed an overall positive contribution as the feature value increased. For the globulin model, 502.456 nm showed a clear negative relationship with model output, whereas 510.491 and 1118.04 nm mainly exhibited positive responses. For the metabolic-protein-sum model, the response patterns of 507.812 and 510.491 nm were similar to those observed in the albumin model, both showing nonlinear changes, whereas 743.526 nm exhibited an approximately monotonic positive contribution.

### 3.9. Cultivar-Grouped Validation of Protein-Fraction Prediction

A leave-one-cultivar-out strategy was used to evaluate the applicability of the ODP-MRMR-RFE-TabPFN framework across different genetic backgrounds. The results of the cultivar-grouped validation are shown in Table 1. Because H1 was the only cultivar included in Experiment 2, its validation results reflect the combined effects of cultivar differences and between-experiment differences. For the held-out H1 group, derivative orders of 1.0, 0.8, and 1.0 were selected for albumin, globulin, and the metabolic-protein sum, respectively, yielding corresponding R^2^ values of 0.639, 0.659, and 0.507.

Across all held-out cultivar groups, the selected derivative orders ranged from 0.8 to 1.2, broadly consistent with the favorable derivative-order range identified in the FOD analysis. For albumin prediction, performance varied substantially among cultivar groups, with R^2^ values ranging from 0.282 to 0.736 and RPD values ranging from 1.180 to 1.945. Only H1, H2, and H3 achieved R^2^ values above 0.630. For globulin prediction, performance was relatively stable across cultivar groups, with R^2^ values ranging from 0.416 to 0.750. The highest accuracy was obtained for J2, with an R^2^ of 0.750, RMSE of 0.926 mg g^−1^, and RPD of 2.001, followed by H1, H2, and H3, with R^2^ values of 0.659, 0.617, and 0.614, respectively. Because the metabolic-protein sum was calculated from albumin and globulin, the stability of its validation performance fell between that of the albumin and globulin models. The R^2^ values ranged from 0.507 to 0.901, with J2, H2, H3, H6, and H7 achieving R^2^ values above 0.630. Overall, the applicability of the ODP-MRMR-RFE-TabPFN framework was influenced by both cultivar and experimental backgrounds, and the magnitude of phenotypic variation appeared to be closely associated with model stability.

## 4. Discussion

### 4.1. Trait-Dependent Predictability of Metabolically Active Protein Fractions

This study showed clear trait-dependent differences in the predictability of wheat metabolically active protein fractions. On the test set, albumin supported quantitative prediction (RPD = 2.554), whereas globulin, which showed a narrower distribution, reached screening-level performance (RPD = 1.804). However, cultivar-grouped validation showed that albumin prediction yielded RPD values ranging from 1.180 to 1.945, indicating that the quantitative predictive performance observed under the random train-test split was not consistently maintained for unseen cultivars. The coefficients of variation were 29.61% for albumin and 14.28% for globulin. Greater target variability provides more informative gradients for spectral regression and may facilitate stable relationships between spectral variation and reference measurements.

Physiologically, albumin participates in grain development, metabolism, and stress responses, so cultivar, water, nitrogen, and fertilization may generate a relatively broad abundance gradient [3]. Globulin may be more genotype-dependent and less responsive to short-term management [4], restricting its calibration range. These observations suggest that the predictability of protein-related traits benefits from sufficient phenotypic variation. Similar difficulty has been reported for individual glutenin subfractions relative to bulk protein-related traits [30]. Broader germplasm, treatment gradients, seasons, and environments may therefore improve calibration for low-variability traits such as globulin.

### 4.2. Order-Dependent Effects of FOD Preprocessing on Spectral Information Enhancement

The model-performance patterns highlighted the importance of treating derivative order as a target- and model-specific preprocessing parameter. For the TabPFN models, ODP identified derivative orders of 1.0 for albumin and 0.8 for globulin. In comparison, the optimal derivative orders identified for the RF models were 1.9 for albumin and 1.3 for globulin. The cultivar-grouped analysis selected derivative orders of 0.8, 1.0, and 1.2, showing that the optimal derivative order could not remain constant when the training-set composition changed. Optimal derivative orders have also varied across application domains, with reported values of 0.6 for soil organic carbon and 1.2–1.6 for canopy chlorophyll [14,15]. These findings suggest that the most suitable preprocessing order may depend on target variability, spectral noise, and model architecture, and the composition of the calibration population. Accordingly, the methodological contribution of this study is the systematic optimization of derivative order within the modeling workflow, rather than evidence for the general superiority of fractional-order differentiation or a universally optimal derivative order. These findings highlight the importance of derivative-order screening in future hyperspectral modeling studies.

### 4.3. Prior-Informed TabPFN for Small-Sample Vis-NIR Spectral Regression

After preprocessing, model architecture influences how effectively the enhanced but still weak spectral information is translated into predictions. The dataset contained only 158 training samples but 268 full-spectrum variables, with strong correlations among neighboring wavelengths, representing a typical small-sample, high-dimensional spectroscopy problem. PLSR handles multicollinearity well but is less flexible for nonlinear or interaction-dependent relationships [16]. SVR captures nonlinear responses but is sensitive to kernel and regularization settings [8], whereas RF may become unstable when many correlated predictors compete for splits under limited sample availability [31]. These limitations do not make conventional models unsuitable, but their bias-variance trade-offs may be less favorable for the present weak-signal dataset.

In this context, TabPFN provides a distinct modeling strategy by incorporating prior information learned from large-scale pretrained tabular task distributions. This prior-informed inference enables efficient prediction with only limited task-specific hyperparameter optimization and allows the model to capture complex nonlinear patterns in small-sample datasets [20]. Furthermore, MRMR-RFE was applied before TabPFN inference to constrain the number of input variables, reduce redundant spectral information, and improve learning efficiency. Therefore, the observed performance should be attributed to the combined effects of MRMR-RFE feature selection and TabPFN rather than to TabPFN alone.

Traditional spectral chemometrics has mainly relied on PLSR, SVR, and tree-based ensemble models, whereas TabPFN has only recently become practically available for regression tasks. Initial applications in spectroscopic food safety and quality assessment, including deoxynivalenol prediction in whole-wheat flour and flavor-related metabolite prediction in meat, suggest that prior-informed learning strategies may provide competitive solutions when chemical reference measurements are expensive and sample expansion is difficult [32,33]. The present study extends this emerging evidence to Osborne protein fractions and demonstrates the potential of prior-informed learning after derivative spectral preprocessing and wavelength selection.

### 4.4. Visible and NIR Features Represent a Coupled Optical Phenotype

The SHAP-based model interpretation revealed differential wavelength dependence among the models for different protein fractions. The visible region contributed the most across the three target models, accounting for 40.6–48.8% of the total contribution. In particular, wavelengths such as 507.812 and 510.491 nm in the 500–590 nm region showed high contributions to the albumin and metabolic-protein-sum models (Figure 11). This result indicates that the visible region contains important predictive information associated with variation in protein fractions.

Visible contributions should not be interpreted as protein-specific absorption. In flour, these features may reflect color, pigments, particle scattering, and starch-protein matrix structure that covary with protein composition [34]. Thus, the identified wavelengths are better regarded as predictive markers than protein-specific absorption bands.

Beyond the visible region, red and NIR wavelengths provided complementary information. Wavelengths around 743 nm contributed positively to the albumin and metabolic-protein-sum models, possibly reflecting variation in flour matrix structure or scattering. NIR wavelengths accounted for more than 36% of the contributions to the globulin and metabolic-protein-sum models. Bands around 1100–1350 nm contain overlapping C-H overtone and N-H/O-H combination information from proteins and other organic constituents while remaining sensitive to matrix scattering [11]. Thus, these models did not rely solely on visible covariance.

SHAP dependence and PDPs further revealed nonlinear, multi-region responses (Figure 12). Albumin showed threshold-like behavior at 507.812 and 510.491 nm; globulin showed a negative response at 502.456 nm and mainly positive responses at 510.491 and 1118.04 nm. TabPFN therefore integrated information across multiple wavelengths rather than relying on a single absorption peak, consistent with contributions from both chemical absorption and physical matrix effects [11].

### 4.5. Methodological Implications, Limitations, and Future Applications

Overall, the ODP-MRMR-RFE-TabPFN framework provides a practical laboratory approach for rapid estimation of metabolically active protein fractions in whole-wheat flour and may support high-throughput comparison of breeding materials and cultivation treatments. Cultivar-grouped validation provided a preliminary assessment of model transferability to unseen genetic backgrounds and indicated that predictive performance was constrained when the model was extended to unseen cultivars.

Nevertheless, several limitations remain in this study. First, the broader applicability of the model should be further enhanced by acquiring samples from different years, ecological regions, and management practices. Second, future studies should explore more compact wavelength subsets or spectral indices to facilitate task-specific multispectral systems. Third, spectra were acquired from whole-wheat flour samples in this study. Although this strategy reduced interference from grain morphology and surface heterogeneity, it also introduced an additional grinding step. Future research should therefore further evaluate the applicability of the proposed framework to intact-grain hyperspectral imaging.

## 5. Conclusions

This study demonstrated the feasibility of Vis-NIR hyperspectral imaging for rapid laboratory-based estimation of metabolically active protein fractions in whole-wheat flour. Albumin showed wider variation than globulin under the tested water-nitrogen and water-fertilizer treatments. The ODP-MRMR-RFE-TabPFN framework was systematically optimized for the three modeling targets, namely albumin, globulin, and the metabolic-protein sum. The corresponding models selected derivative orders of 1.0, 0.8, and 1.0, retained 147, 46, and 70 wavelengths, and achieved test-set R^2^ values of 0.847, 0.693, and 0.850, respectively. Model interpretation showed that visible wavelengths contributed strongly, whereas retained NIR wavelengths supplied complementary information. Independent re-sampling validation supported reproducibility of separate sample preparation and measurement within the same field harvest but did not constitute external biological validation. Leave-one-cultivar-out validation showed heterogeneous cross-cultivar transfer. Albumin R^2^ values ranged from 0.282 to 0.736, whereas globulin R^2^ values ranged from 0.416 to 0.750. Overall, the framework is a promising laboratory-based strategy for phenotyping wheat flour protein fractions, and its broader applicability should be further established by including a wider range of cultivars.

## Figures and Tables

**Table 1:** Leave-one-cultivar-out grouped validation performance for ODP-MRMR-RFE-TabPFN models.

Analyte	Metric	Held-Out Cultivar					
		H1	J2	H2	H3	H6	H7
albumin	Order	1.0	1.0	1.0	0.8	1.0	1.0
	Variables	120	86	27	182	38	72
	R^2^	0.639	0.541	0.677	0.736	0.302	0.282
	RMSE	2.696	1.417	1.829	1.563	1.156	1.440
	RPD	1.663	1.476	1.760	1.945	1.197	1.180
globulin	Order	0.8	0.8	1.0	1.2	1.2	1.2
	Variables	83	157	126	142	246	17
	R^2^	0.659	0.750	0.617	0.614	0.454	0.416
	RMSE	0.960	0.926	1.057	0.937	0.544	0.803
	RPD	1.711	2.001	1.615	1.609	1.353	1.308
metabolic-protein sum	Order	1.0	1.0	1.0	0.8	0.8	0.8
	Variables	52	78	47	96	209	197
	R^2^	0.507	0.901	0.761	0.860	0.639	0.648
	RMSE	3.182	1.006	1.794	1.434	1.014	1.143
	RPD	1.424	3.185	2.046	2.674	1.664	1.687

Note: Variables = number of input variables; RMSE values are expressed in mg g^−1^.

## Data Availability

The original contributions presented in this study are included in the article. Further inquiries can be directed to the corresponding authors.

## Appendix A

**Table A1:** Comparison of model performance for albumin, globulin, and the metabolic-protein sum using different variable selection methods.

Analyte	Set	Metric	Variable Selection Strategy				
			Full	MRMR-RFE	UVE	CARS	SPA
albumin	CV	R^2^ mean	0.715	0.726	0.548	0.579	0.429
		RMSE mean	3.717	3.655	4.721	4.510	5.347
		RPD mean	1.981	2.055	1.538	1.642	1.343
	Test	R^2^	0.834	0.847	0.699	0.693	0.558
		RMSE	3.200	3.073	4.305	4.349	5.218
		RPD	2.452	2.554	1.823	1.805	1.504
globulin	CV	R^2^ mean	0.465	0.481	0.420	0.487	0.400
		RMSE mean	1.357	1.334	1.419	1.319	1.424
		RPD mean	1.405	1.432	1.347	1.444	1.342
	Test	R^2^	0.650	0.693	0.479	0.567	0.497
		RMSE	1.015	0.951	1.238	1.129	1.217
		RPD	1.691	1.804	1.385	1.520	1.410
metabolic-protein sum	CV	R^2^ mean	0.717	0.706	0.691	0.722	0.691
		RMSE mean	4.119	4.220	4.319	4.075	4.298
		RPD mean	2.083	2.021	1.981	2.105	1.996
	Test	R^2^	0.815	0.850	0.720	0.830	0.827
		RMSE	3.750	3.368	4.611	3.587	3.627
		RPD	2.323	2.586	1.889	2.428	2.401

Note: CV = mean performance of five-fold cross-validation; Test = independent test set performance; RMSE values are expressed in mg g^−1^.
